# Grazing Ecology of Sheep and Its Impact on Vegetation and Animal Health in Pastures Dominated by Common Ragwort (*Senecio jacobaea* L.)—Part 1: Vegetation

**DOI:** 10.3390/ani12081000

**Published:** 2022-04-12

**Authors:** Susanne Ohlsen, Martin Ganter, Peter Wohlsein, Bernd Reckels, Aiko Huckauf, Nikola Lenzewski, Sabine Aboling

**Affiliations:** 1 Institute of Animal Nutrition, University of Veterinary Medicine Hannover, Foundation, 30173 Hannover, Germany; bernd.reckels@tiho-hannover.de; 2 Clinic for Swine, Small Ruminants, Forensic Medicine and Ambulatory Service, University of Veterinary Medicine Hannover, Foundation, 30173 Hannover, Germany; martin.ganter@tiho-hannover.de; 3 Department of Pathology, University of Veterinary Medicine Hannover, Foundation, 30559 Hannover, Germany; peter.wohlsein@tiho-hannover.de; 4 Nature Conservation Foundation Schleswig-Holstein, 24113 Molfsee, Germany; aiko.huckauf@stiftungsland.de; 5 Institute of Plant Science and Microbiology, Universität Hamburg, 22609 Hamburg, Germany; nikola.lenzewski@uni-hamburg.de

**Keywords:** *Senecio jacobaea* L., sheep, grazing behavior, animal health, free-choice conditions, ecological impact, conservation grazing

## Abstract

**Simple Summary:**

Common ragwort (*Senecio jacobaea* L.) is a worldwide established plant containing toxic pyrrolizidine alkaloids (PA), which can lead to liver cirrhosis in livestock, especially cattle and horses. Controlling ragwort, particularly in conservation areas, is difficult. As sheep seem more resistant to PA, sheep might be a natural defense against the observed spread of ragwort. We tested this in a two-year study on a ragwort-rich pasture with 12 sheep/hectare from May to October 2020–2021. We addressed three questions: (1) To what extent do sheep voluntarily ingest ragwort? (2) In what respect do feeding behavior and nutritional parameters correlate? (3) What impact does grazing have on the yield proportion and number of flowers of dominant plants? We showed that sheep preferred ragwort without any harmful effects. The more ragwort was available and the more it contained sugar, the higher the amount ingested by the sheep. Ragwort accounted for a significantly lower yield proportion of ingested biomass in the second year even though its number of flowers doubled. The yield of biomass of other herbs increased. From the point of view of animal health and nature conservation, sheep grazing on ragwort might be an option to reduce the amount of ragwort in pastures.

**Abstract:**

Species-rich pastures naturally contain potentially toxic plants such as common ragwort (*Senecio jacobaea* L.), whose pyrrolizidine alkaloids (PA) impose a risk, mainly for cattle and horses. Although in vitro studies showed detoxification capacity of PA in sheep, few field data are available to ascertain whether grazing sheep can both tolerate and reduce ragwort. In a two-year study in a ragwort-rich pasture with a stocking density of 12 sheep/hectare, we documented (1) the extent of voluntarily ingested ragwort, (2) the correlation of nutritional parameters and feeding behavior, and (3) the impact of grazing on the yield proportion and number of flowers of dominant plants. Every six weeks the vegetation underwent a botanical survey and a chemical analysis. Sheep continuously ingested ragwort between 1.2 and 4.9 kg (2020) and 1.0 and 2.2 kg (2021) per individual per day without any impact on animal health. The more biomass ragwort produced, the more it contained sugar (*r* = 0.59–0.74), and the more sheep ingested it (*r* = 0.94–0.95). Other herbs increased their yield proportion from 23.3 to 36.5%, while that of ragwort decreased from 26.3 to 18.8% (2020/2021), doubling its flowers. Sheep preferred and tolerated ragwort, making their grazing an option to control ragwort from both an animal health and a nature conservation perspective.

## 1. Introduction

Animal experiments [1] and food scarcity [2,3,4,5,6,7,8,9,10,11,12,13,14] have proven the toxicity of pyrrolizidine alkaloids, typical for common ragwort (*Senecio jacobaea* L.), in livestock animals. However, field studies revealed that large herbivores may cope well with toxic plants, provided they are socialized and enjoy free choice [15]. There are successful examples of farmers in the USA who use sheep to reduce ragwort populations by exploiting the animal species’ high tolerance and detoxification ability [9,16,17,18] due to the fact that the rumen biome is able to eliminate pyrrolizidine alkaloids to a limited extent [9,19,20,21]. In these cases, sheep grazing was recommended as a biological control agent for the weed [9,22,23,24,25], albeit for one season only [9]. In many countries, environmentally friendly options are needed to reduce common ragwort alongside cutting it by hand, as recommended in several studies [26,27,28,29,30]. Eradicating ragwort via biocides leads to a decrease in biodiversity [31], while sheep would be ideal partners in the natural control of ragwort. However, apart from open questions on possible health risks, sheep may cause ecological damage by reducing the number of flowers [31]. In our field study on a ragwort-dominated pasture, we not only provided freedom of choice for a ragwort-tolerant animal species but also addressed the problem whether significant ecological parameters would be influenced by grazing. Three questions were posed: (1) To what extent do sheep ingest ragwort? (2) In what respect are feeding behavior and nutritional parameters correlated? (3) What impact does grazing have on the yield proportion and number of flowers of dominant plants?

## 2. Materials and Methods

### 2.1. Project Area

The project area “Stellmoorer Tunneltal” (Google maps coordinates: 53.618254, 10.179990 or 53°37′05.7″ N 10°10′48.0″ E) is a semi-open pasture and highly visitor-frequented nature reserve of the metropole Hamburg in Northern Germany. Its species-rich ancient grassland is almost exclusively pastured by cattle with a stocking density of 0.3–0.5 adult cattle per hectare. As it is a nature conservation area, mowing and fertilizing of the pastures are not allowed by German law.

### 2.2. Study Design

**Figure 1:**
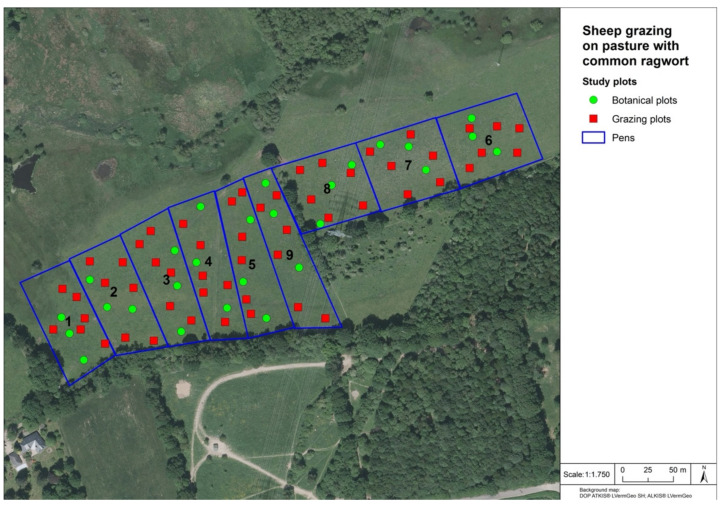
Schematic classification of the nine pens (blue lines, numbers 1 to 9), 54 grazing plots (red markers), and 27 botanical plots (green markers). (Background map: DOP ATKIS^®^ LVermGeo SH; ALKIS^®^ LVermGeo).

At the beginning of the project, 70 sheep (White Polled Heath sheep and their cross breeds), consisting of 16 animals older than one year and 54 gimmers, were purchased. Out of this group, seven sheep were randomly picked and slaughtered as a first control group. The remaining 63 sheep were placed in a fenced area of 5.25 hectares divided into nine pens of 0.58 hectares each, corresponding to a stocking rate of 12 sheep/hectare (Figure 1). In each pen, nine 20 m^2^ plots of 4.47 m × 4.47 m were randomly chosen and installed: (1) six plots for monitoring the grazing impact (grazing plots, 54 in total in the whole area), e.g., counting the ingested ragwort plant parts, (2) three plots for botanical monitoring and sampling (botanical plots, 27 in total in the whole area), e.g., determination of plant species, estimation of yield proportions as well as taking vegetation samples for analysis. Hay from a hay rack and sheep mineral feed were provided ad libitum.

The field study comprised two grazing seasons from 4 May until 21 October 2020 and from 3 May until 14 October 2021. Every six weeks (grazing period), seven sheep were randomly selected and slaughtered, and the grazing area was reduced by one pen (first pen no. 1 followed by pen no. 2 and so on) (Figure 1) to maintain a continuous stocking density of 12 sheep/hectare. At the end of the first grazing season (May–October 2020), the remaining 35 sheep were removed from the study area and kept on a ragwort-free winter pasture elsewhere. In the second grazing season (May–October 2022), the process was repeated, i.e., seven sheep were randomly selected and slaughtered as a second control group, and the remaining 28 animals started grazing on a pasture that consisted of four pens (numbers 6–9) only.

### 2.3. Plant Sampling and Vegetation Survey

For analysis of nutritional parameters, vegetation samples were taken in the beginning and at the end of each grazing period from “active” pens, where sheep were still grazing. Each time, three vegetation samples of 1 m^2^ each were harvested next to the botanical plots of the respective pen, and a sample of fresh ragwort (one sample of 500 g) was randomly harvested in the same pen. The biomass was cut as close to the ground as possible using electric and mechanical grass shears. The samples were placed in plastic bags, which were then sealed and transported overnight to the laboratory for immediate analysis.

The analyzed parameters included fiber and protein (Weende analysis, described in [32]) as well as sugar and macro- and microelements.

Additionally, vegetation data were collected in both botanical and grazing plots. In the botanical plots, we collected the following data at the beginning and at the end of each grazing period (six-week intervals): (1) biodiversity (number of plant species per plot along with their abundances according to the Londo method [33]); (2) number of flowers per botanical plot, estimated in classes of *n* = 1–5, 6–10, 11–20, >20, >50, and >100; (3) yield proportions of fresh grasses, other herbs, and ragwort according to E. Klapp cited in [34], e.g., estimating the percentage of the harvestable aboveground plant parts, in our case, of fresh ragwort, other herbs, and grasses.

In the grazing plots, we also estimated, at two-week intervals, yield proportions of fresh ragwort, other herbs and grasses, also according to E. Klapp, (parallel to the counting of ingested ragwort, see Section 2.4).

Nomenclature of plant species followed Schmeil–Fitschen [35]. Species of the genus *Poa* were summarized to *Poa spec.* and were treated as one species in the analysis.

### 2.4. Monitoring Ragwort Grazing Behavior 

Grazing behavior was documented as the intake of ragwort plant organs to be seen as missing plant parts, assumed to be ingested by the sheep due to typical traces of ruminant grazing. Monitoring took place on a two-week basis by counting missing full and half leaves of fresh ragwort rosettes and later also shoots, flower buds, shoots, and stems during both grazing seasons from May until October 2020 and 2021. The weight of the missing ragwort parts in grams was calculated by weighing reference plant material. For this purpose, we collected full and half ragwort leaves from the pasture three times and took the average value. Furthermore, we used reference plants, also randomly harvested in the pasture, artificially defoliated them according to the pattern we observed in the pasture and weighed the plant parts in distinct states of defoliation.

Missing ragwort plant parts were counted, as described above, providing data on both the kind and amount of ingested ragwort parts relevant for the feeding behavior. Due to the frequent monitoring, destroyed or otherwise disappeared ragwort leaves could be detected. Shoots grazed partly and repeatedly grazed may have been counted erroneously several times. To correct this, we divided the total number of browsed shoots counted within a grazing period (six weeks) by the number of counts of the corresponding grazing period. At the beginning of July 2020, when mainly long ragwort shoots were left, being defoliated by the sheep, the counting of ingested full and half leaves on these shoots proved impossible due to their large number of partially up to more than one thousand shoots per plot. Therefore, we estimated the missing (ingested) full and half leaves of the shoots from the beginning of July until mid-October 2020 as a missing percentage of the original biomass of ragwort leaves and shoots, i.e., how much (in percent) of the original ragwort plant was missing. We converted the estimated percentages of missing full and half ragwort leaves with our data from the botanical plots, using the weight of the harvested biomass (same grazing period) from the botanical plots (1 m^2^) and the estimated amounts of yield proportions (ragwort, other herbs, and grasses were separately estimated). To obtain comparable numbers, we calibrated the estimated percentages according to the estimated yield proportions (ragwort, other herbs, and grasses) using the weight of the harvested biomass from the botanical plots (1 m^2^).

### 2.5. Statistical Analysis

The statistical analysis was performed with the calculation programs of Microsoft Office, Microsoft Office 2016^®^ and the SAS^®^ Enterprise Guide^®^, version 7.1 (SAS Institute Inc., Cary, NC, USA).

Two-tailed paired sample t-tests were used after testing for a normal distribution. Correlation was calculated using the Pearson coefficient. Statistical results were assumed to be significant at a level of *p* < 0.05.

## 3. Results

### 3.1. Extent of Ragwort Intake

**Figure 2:**
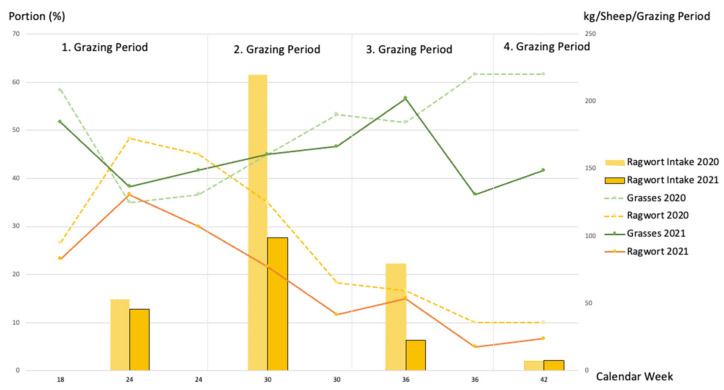
Average yield proportions of grasses and ragwort (lines; units left ordinate) vs. ingested ragwort (bars; units right ordinate) in kg (OS) per sheep and grazing period (=45 days = six weeks) in 2020 and 2021. Data obtained from grazing plots.

In the first year, sheep began ingesting ragwort during the first six weeks (1st grazing period), while ragwort still managed to increase its average yield proportion in the grazing plots from 26.6% to 45.0%. At the same time, the average yield proportion of grasses dropped from 58.3% to 36.6% (Figure 2).

Sheep started ingesting full and half leaves of ragwort first from the rosettes and when developed also from the shoots (9.37 kg original substance (OS)/sheep/grazing period) during the first grazing period of the first year; overall, however, they ingested more shoots (44.1 kg OS) (Table 1).

The animals ingested even more ragwort shoots and flower buds (123 kg OS) during the second grazing period, starting with the uppermost parts and ending 10 to 15 cm above the ground in most of the cases. They also ingested a fair number of leaves from rosettes and shoots (97.1 kg OS), which resulted in a maximum overall ragwort intake of 220 kg OS/sheep. During this second grazing period, the average yield proportion of ragwort decreased from 35.0% to 18.3%, whereas that of grasses continuously increased from 45.0% to 53.3%. Within the third and fourth grazing periods of the first year, the mean ragwort intake per sheep dropped onto 79.5 kg OS and 7.28 kg OS, respectively. In these periods, the average yield proportions of ragwort dropped from 16.6% to 10.0%, and the average yield proportions of grasses slightly decreased to 51.6% and then slightly increased again to 61.6%.

In the second year, the sheep displayed the same feeding pattern, albeit at a lower level. Surprisingly, the 28 remaining sheep ingested almost the same total number of half and complete leaves (*n* = 20,847.5 = 15.36 kg OS) in the first grazing period as the complete flock of 63 sheep at the beginning of 2020 (*n* = 16,507.5 = 12.14 kg OS). During this first grazing period of 2021, the average yield proportion of ragwort continuously decreased from 36.6% to 30.0%. In contrast, grasses continuously increased from 38.3% to 41.6%. Likewise, during the second and third grazing periods, sheep reduced their mean intake of ragwort per individual from 98.9 kg OS (compared to 220 kg OS in the first year) to only 22.6 kg OS (79.5 kg OS in the first year), although there was no significant statistical effect throughout the whole season (*p* = n.s.). The yield proportion of ragwort further decreased during the second grazing period from 21.6% to 11.6% and during the third period from 15.0% to 5.0%. The yield proportions of grasses hardly changed during the second period (from 45.0% to 46.6%) but dropped at the end of the third period from 56.6% to 36.6%. In contrast to the first year, sheep began to ingest shoots with undeveloped flower buds on young stalks.

### 3.2. Nutritional Value of Ragwort Compared to Other Plants

**Figure 3:**
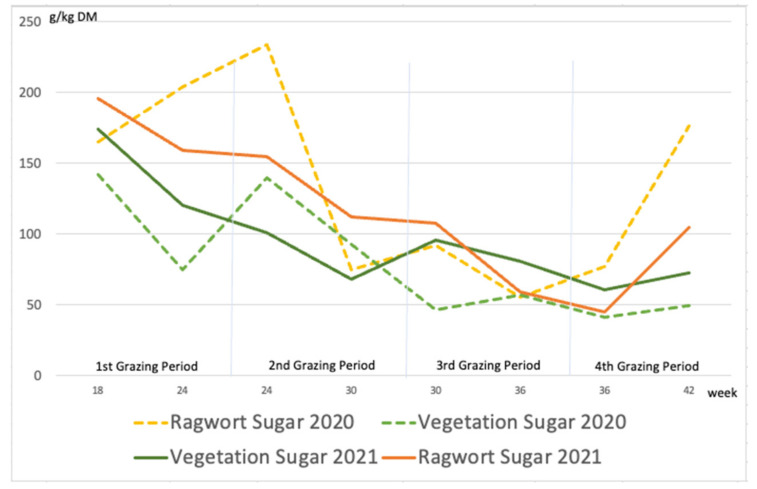
Content of sugar (g/kg DM) in ragwort and other plants (“vegetation”) over the course of the four grazing periods in 2020 and 2021.

The content of sugar (g/kg dry matter (DM)) as a highly digestible nutrient showed the same seasonal variation in ragwort as in other plants (“vegetation”) (Figure 3).

The mean sugar content in ragwort was 135 and 117 g/kg DM in the first and second year, respectively, and thus higher than that in the remaining vegetation (80.4 and 96.6 g/kg DM; *p* = 0.07 and *p* = n.s.), (Table 2). In 2020, the mean sugar content in ragwort (134 ± 63.4 g/kg DM) was only slightly higher than that in 2021 (117 ± 47.7 kg/kg DM; *p* = n.s.).

**Figure 4:**
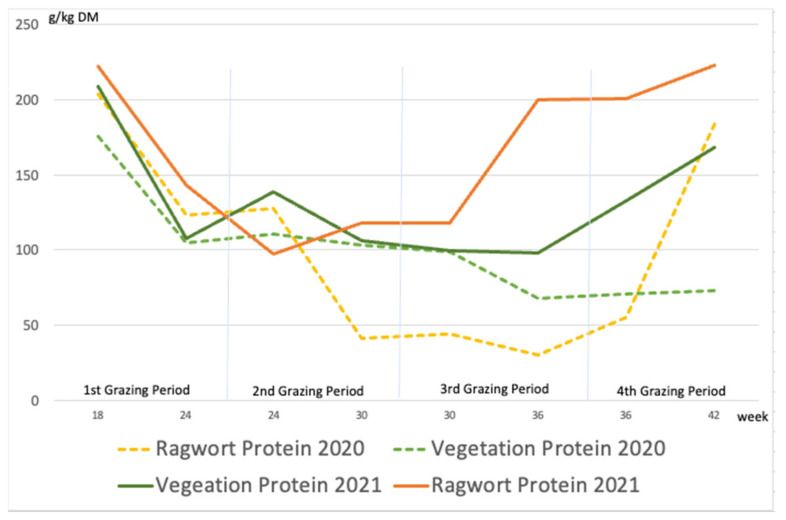
Content of crude protein (g/kg DM) in ragwort and other plants (“vegetation”) during the experiment.

Whereas ragwort and other plants showed similar protein contents at the beginning of grazing, this nutrient was less concentrated in ragwort than in the rest of the vegetation during the third grazing period of the first year (Figure 4). In contrast, ragwort contained higher amounts of protein than the rest of the vegetation throughout most of the second year. Both were due to the fact that in the first year, the ragwort samples mainly contained stalks, which are typically poor in protein, whereas in the second year, the samples contained no such organs because sheep had eaten the young shoots from the beginning of the second year (see end of Section 3.1). Thus, the ragwort samples from the second year were composed of slender and young stems containing more protein than thick and adult ones.

**Figure 5:**
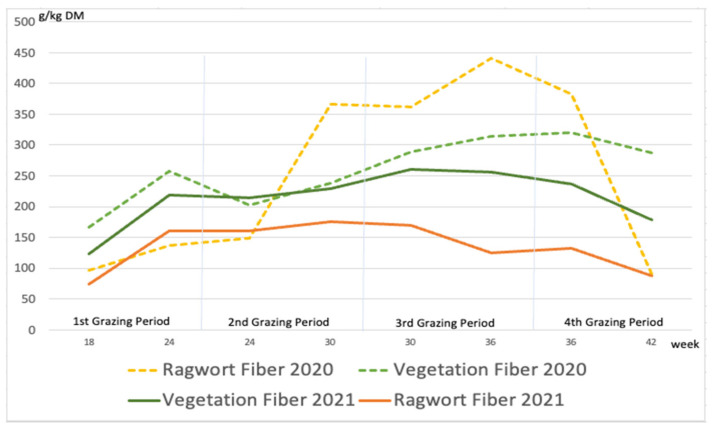
Crude fiber content (g/kg DM) in ragwort and other plants (“vegetation”) during the experiment.

As usual and in contrast to sugar, the crude fiber content was highest towards the end of the season (Figure 5). In the first year, the mean fiber content in ragwort (253 g/kg DM) and other plants (259 g/kg DM) was similar (Figure 5). Unlike the sugar content, which was on average only slightly higher in ragwort than in other plants, the mean fiber content in ragwort (135 g/kg DM) was significantly lower than that in the rest of the vegetation (215 g/kg DM; *p* < 0.05).

### 3.3. Correlation of Feeding Behavior and Nutritional Parameters

To study the relationship between the feed quality of ragwort and the rest of the vegetation, we concentrated on selected nutritional parameters on the one hand and the mass intake of ragwort as well as the yield proportions of ragwort and other plants on the other hand. We found a positive correlation between the sugar content and biomass proportion in ragwort in both years (*r* = 0.59 and 0.74, respectively), whereas there was no such correlation in other plants (“vegetation”) (*r* = −0.41 and 0.31; Table 3).

The more sugar ragwort contained in both years (*r* = 0.86 and *r* = 0.65, respectively), the more sheep ingested it (Table 4). Sheep ate ragwort when it was available in the first year (*r* = 0.94). However, this was not the case in the second year (*r* = 0.45) when both the yield proportion and intake of ragwort were overall reduced (see Figure 2).

### 3.4. Impact of Sheep Grazing on Yield Proportion and Number of Flowers of the Vegetation

**Figure 6:**
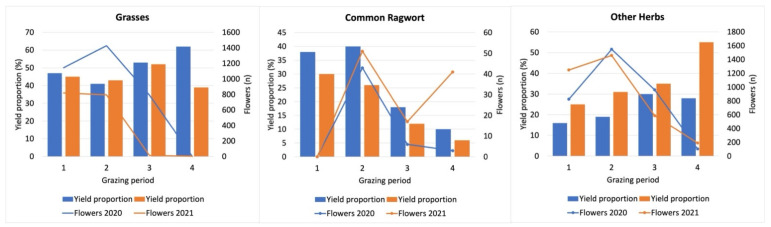
Mean yield proportions (in percent; units left ordinate) and flowers (*n*; right ordinate) of common ragwort, other herbs, and grasses. Data obtained from botanical plots.

Sheep grazing influenced the vegetation in several respects. Similar to the results for the grazing plots described above (Figure 2), the average yield proportion of ragwort in the botanical plots decreased from 38.0% to 10.0% in the first year and from 30.0% to 6.0% in the second year (Figure 6). As described for the grazing plots, portions of all other herb species tended to increase from 16.0% to 28.0% in the first year and from 25.0% to 55.0% in the second year. Yield proportions of grasses oscillated between 47.0% and 62.0% in 2020 and 45.0% and 39.0% in 2021. However, the mean yield proportions of grasses in the grazing plots (50.4 and 44.8%) were higher than those of ragwort (26.3% and 18.8%) (*p* < 0.005 and *p* < 0.001) in both years, whereas other herbs (36.5%) had higher portions than ragwort (18.8%) only in the second year (*p* < 0.05).

The individual yield proportions correlated only partly with each other. For example, the yield proportions of other herbs increased, whereas the yield proportions of ragwort decreased in both years (*r* = −0.81 and *r* = −0.85, respectively; Table 5). Interestingly, the yield proportions of grasses increased at the expense of ragwort in the first year (*r* = −0.93), but no correlation could be found in the second year (*r* = −0.05) when the yield proportions of grasses oscillated more erratically.

Regarding the relationship between yield proportions and number of flowers, there were distinct differences between the plant groups and years. As for ragwort, four phenomena could be observed: First, its yield proportions tended to be smaller in the second year. Second, there were 1736 fewer main flowering shoots in the second year. Third, the ragwort population developed 132 side shoots in the second year as opposed to none in the first year. Fourth, compared to the first year with 52 flowers in the first four pens (numbers 1–4), in the second year, there were 109 flowers in the second four pens (numbers 6–9; Table 6). In contrast, the yield proportions of grasses did not change significantly in both years, yet their number of flowers decreased significantly from 3365 in the first to 1632 in the second year. Although the yield proportions of other herbs were continuously higher in the second year (Figure 6), their number of flowers remained almost the same in both years (Table 6).

**Figure 7:**
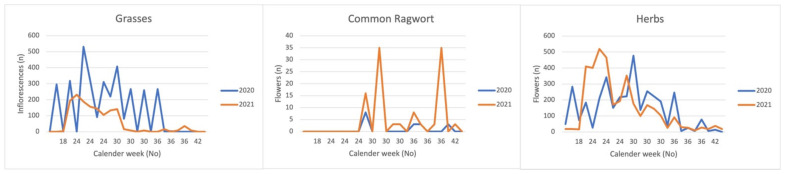
Number of flowers of ragwort, other herbs, and grasses in both years per calendar week.

Overall, in ragwort as well as in both grasses and other herbs there was a second stronger flower interval during the 30th week in the second year (Figure 7). Ragwort also showed an additional third interval during the 36th week.

### 3.5. Plant Species Diversity and Vegetation Composition

During the two grazing periods of 2020 and 2021, a total of 51 different plant species were recorded with no differences between the years (mean values across all botanical plots: 22 in 2020 and 21 in 2021). Grasses such as *Agrostis stolonifera* and *Lolium perenne* as well as other herbs such as *Senecio jacobaea* and *Cirsium arvense* were the most abundant species (Table 7). As for the grasses, we noticed a significant decline in the number of *Agrostis capillaris*, *Alopecurus pratensis*, *Anthoxantum odoratum*, *Bromus hordeaceus*, *Cynosurus cristatus* and *Poa spec.*, whereas *Holcus lanatus* and *Lolium perenne* slightly increased in number. *Lolium perenne*, which is supposed to be stimulated by common ragwort according to Ahmed and Wardl, (1994) cited in [36], increased from 2020 to 2021. However, ragwort proportions in 2021 (mean = 18.8%) always remained below those of 2020 (mean = 26.3%; *p* = n.s.).

The coverage of grass species slightly changed from the first to the second year. The species composition of the most abundant grasses differed only for four species. *Carex hirta* and *Agrostis stolonifera* were replaced by *Festuca rubra* and *Dactylis glomerata* in the top 10 list in 2021. Minor variations also applied to the coverages of herbs; here, *Potentilla anserina*, *Achillea millefolium*, *Hypochaeris radicata*, and *Veronica serpyllifolia*, were replaced by *Potentilla anserina*, *Achillea millefolium*, *Hypochaeris radicata*, and *Veronica chamaedrys* in the top 10 list.

Most noticeable was the increase in *Cirsium arvense*, which almost doubled from an average coverage of 8.0% to 15.0% within one year.

## 4. Discussion

The aim of the study was to investigate (1) the extent of voluntarily ingested ragwort, (2) the relationship between nutritional parameters and feeding behavior, and (3) the impact of grazing on yield proportions and number of flowers of dominant plants.

### 4.1. Extent of Voluntarily Ingested Ragwort and Relationship to Nutritional Parameters

Even though in previous studies sheep either had to be taught to ingest common ragwort or even refused it [37,38], our data proved that there were sheep that readily ingest substantial amounts of ragwort from the first day to the last under free-choice conditions. Not only was the yield proportion of grasses twice that of ragwort, but other herb species had similar proportions (2020) and in fact doubled their share (2021). Since there was more alternative feed than ragwort available, voluntary intake of such large quantities of ragwort is an indication of ragwort preference [39]. Moreover, since the sugar content and ragwort intake were strongly correlated in both years, ragwort turns out to be a feed that is favored due to its palatability or energy content. Particularly in the second year, ragwort was more attractive than other plants; first, due to its protein content, which turned out to be similar to or even higher than that of grasses, and second due to its slightly (first year) to significantly (second year) lower fiber content. In conclusion, we found strong indications that sugar was revealed to be the decisive variable that makes ragwort highly attractive and led to its being extensively consumed at least in the first year, whereas in the second year, its higher protein content might have increased its appeal. Especially in summer, at times of dry feed, ragwort turned out to be a nutritional and valuable food for sheep [18,26,29]. Furthermore, ragwort is rich in sodium, chlorine, and copper (Fairburn and Thomas, 1959, cited in [40].

In addition, buds, the most energy-rich plant organ, contribute to the palatability of fresh ragwort. The so-called apical grazing ([41], not published) observed in browsing goats might also be an adapted grazing behavior in sheep under free-choice conditions. Since buds constitute a small biomass proportion among other available plant parts such as leaves, this means not only feed preference but also feed selection [42]. Our analyzed data confirm such selection behavior: despite striking differences in the absolute figures for the two years, the sugar content of ragwort and its intake peaked simultaneously in both years of the study. Since the sugar content and yield proportions of ragwort were also strongly correlated (*r* = 0.70 and 0.97 in 2020 and 2021, respectively), this resulted in a high intake of ragwort and contributed to the desired effect of its reduction as a positive effect (see below).

From the point of view of animal health, palatable ragwort stimulates sheep to eat more of it under free-choice conditions than experimental conditions revealed to be tolerable. The sheep in our study each ingested between 0.16 and 4.89 kg ragwort per day (Table 1), exceeding the supposed lethal doses of common ragwort in sheep. Surprisingly, our data prove no hints to periods of adaptation [43] or negative post-ingestion feedback [44]. On the contrary, sheep ingested more ragwort the more palatable it was and the more available it became. Since in our study the proportion of grasses and other herbs never dropped below 50% (yield proportions), the sheep did not resort to ragwort due to a lack of alternative food at any time. This proves that they ingested it voluntarily.

### 4.2. Impact of Grazing on Yield Proportions of Dominant Plants

**Figure 8:**
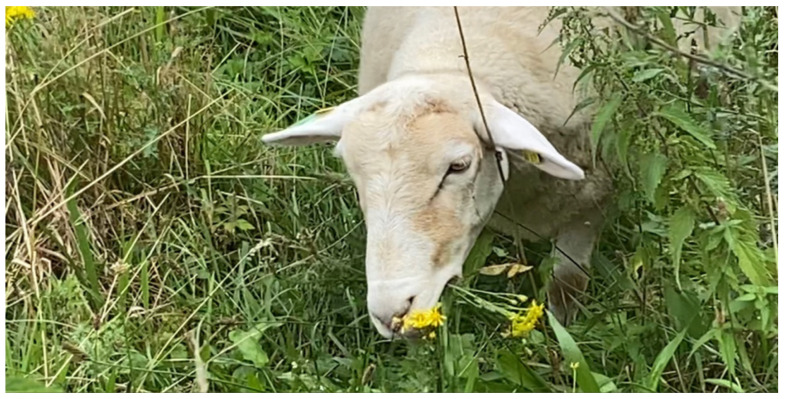
Sheep grazing ragwort flower in a rich pasture. (Date: 9 August 2021).

**Figure 9:**
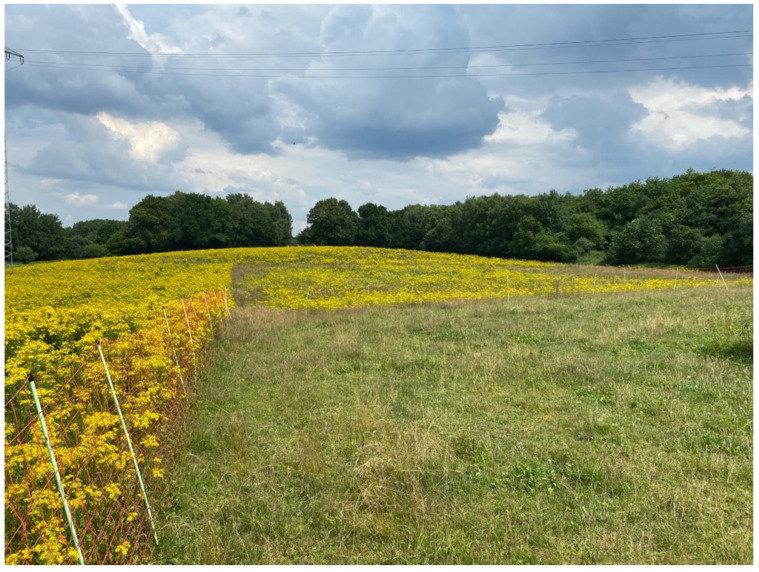
Left of the fence: pasture grazed by cattle only; in the background: pasture grazed by sheep six weeks earlier (ragwort grew back and flowered, but was only about half its usual height, less dense, and clearly reduced); in the foreground: area currently grazed by sheep. (Date: 12 July 2021).

Feeding preferences influence the vegetation structure, in particular ragwort itself. Due to the preference for generative organs (Figure 8) (flowers and apical parts of whole inflorescences in the first year as well as buds and young stems in the second year), sheep markedly change the morphology in relation to the habitus of ragwort. In contrast to the first year, sheep began to eat shoots with undeveloped flower buds on young shoots in the second year (cf. Section 3.1). This prevented the plants from developing a strong main shoot. Moreover, the damage in the first year left the plants with a reduced leaf area so that they could not produce as many carbohydrates as before grazing. Along with the early removal of the buds in the second year, in which no main shoots but numerous slender and leaf-rich stems were observed, the plants switched to a generative mode in which they strived to regenerate themselves. In the second year, this resulted in a high number of flowers produced by multi-stemmed plants with multiple flowers within only six weeks after the sheep had been removed from the respective pen (Figure 9).

A higher nutritional value in ragwort than in the other plants could explain why the lesser nutritional other herbs (analyzed chemically together with grasses) were not grazed at a similar intensity and thus could increase their mean proportions from 23.3% to 36.5% from the first to the second year, eventually exceeding the proportions of ragwort in the second year (18.8%). However, certain herbaceous species showed a decline, too, such as *Trifolium repens* and *Plantago lanceolata*, which are quite palatable for sheep [45,46]. Along with most feed plants, the avoided weed *Cirsium arvense* increased its coverage from 7.63% to 14.5% during the two-year period.

### 4.3. Impact of Grazing on Numbers of Flowers

While both ragwort and other herbs increased their yield proportions in the second year, only the numbers of flowers of ragwort increased, whereas those of other herbs remained unaffected and those of grasses decreased. During grazing, there were almost no ragwort flowers visible. However, ingestion of flower buds did not suppress the development of blossoms in general. On the contrary, mechanical damage along with a reduction in biomass stimulated a rapid regeneration during grazing. As already observed by Sharrow et al. (1980) [18], damaged ragwort plants are well adapted to grazing and produce numerous slender flowering side shoots. In our study, this resulted in an increased number of flowers in the second year, a typical ecological answer to balance losses in offspring.

The fact that the inflorescence of grasses was halved in 2021 is probably due to sheep grazing. The biomass of grasses and ragwort was more or less negatively correlated. Thus, the reduction in ragwort led to an increase in the vegetative regeneration of the biomass of grasses; however, sheep might have selected the most apical growing and nutritious plant organs such as flowers and unripe seeds by applying so-called apical grazing ([41], not published). In contrast, there was no suppression of flower formation in other herbs as an undesired collateral effect of grazing as observed by Crawley [31]. Instead, we observed not only an increase in the yield proportions in that plant group, but also a slightly longer lasting bloom in the second year.

## 5. Conclusions

Under free-choice conditions, sheep clearly possess the ability to ingest significantly larger amounts of common ragwort than the currently assumed lethal doses [25,47,48,49]. Ragwort, known mainly for its toxic PA and regarded as a general hazard, turned out to be not only tolerable at least for sheep, but also more palatable than all other plants in the pasture. Furthermore, sheep were able to significantly reduce the ragwort population without damaging the overall number of flowers. However, we still do not know which strategy naïve sheep used to cope with the dominating ragwort under the conditions we offered. Moreover, we did not address the question of what kind of grazing management concerning stocking rate and grazing duration is optimal to sustainably reduce ragwort. Nevertheless, sheep grazing appears to be both an animal and environmentally friendly option to control common ragwort in sensitive grasslands, e.g., in nature conservation areas.

## Figures and Tables

**Table 1:** Total ingested ragwort biomass in kg per sheep and per grazing period (45 days), also calculated as ragwort biomass in kg per sheep and day; furthermore, the amounts of ingested leaves (from rosettes and shoots) and shoots are shown in kg per 45 days (grazing period).

Grazing Period No./Year	Biomass (kg/Sheep/45 d)	Biomass (kg/Sheep/d)	Leaves (kg/45 d)	Shoots (kg/45 d)
1/2020	53.5	1.19	9.37	44.1
2/2020	220	4.89	97.1	123
3/2020	79.5	1.77	32.6	46.8
4/2020	7.28	0.16	7.17	0.11
1/2021	45.8	1.02	26.6	19.2
2/2021	98.9	2.20	21.1	77.7
3/2021	22.6	0.50	7.14	15.5
4/2021	7.49	0.17	7.18	0.30

**Table 2:** Comparison of nutritional parameters in ragwort and vegetation (mean values ± standard deviation) with *p* values according to two-sided paired t-tests. n.s. = not significant.

	Mean Content/g/kg DM					
	2020			2021		
	Sugar	Protein	Fiber	Sugar	Protein	Fiber
**Ragwort**	135 ± 63.4	101.2 ± 63.9	253.0 ± 138.0	117.0 ± 47.7	165.0 ± 48.2	135.0 ± 35.7
**Vegetation**	80.4 ± 38.4	101.0 ± 32.7	259.0 ± 50.9	96.6 ± 34.4	132.0 ± 36.6	215.0 ± 41.8
***p***	0.07	n.s.	n.s.	n.s.	n.s.	<0.01

**Table 3:** Matrix of yield proportions and nutritional parameters in ragwort and other plants (“vegetation”).

Nutritional Parameter (g/kg DM)	Yield Proportion (%)			
	Ragwort		Vegetation	
	2020	2021	2020	2021
Ragwort, fiber	−0.37	0.33	-	-
Ragwort, sugar	0.59	0.74	-	-
Vegetation, fiber	-	-	0.33	−0.06
Vegetation, sugar	-	-	−0.41	0.31

**Table 4:** Matrix of ragwort intake and selected variables.

Year of the Ragwort Intake	Yield Proportion (%)		Content (g/kg DM)			
			Sugar		Fiber	
	Ragwort	Grasses	Ragwort	Grasses	Ragwort	Grasses
2020	0.94	−1.0	0.86	0.59	−0.49	−0.47
2021	0.45	0.13	0.65	0.30	0.14	−0.28

**Table 5:** Correlation matrix of yield proportions in ragwort, other herbs, and grasses.

Yield Proportion (%)	Yield Proportion (%)			
	Ragwort		Grasses	
	2020	2021	2020	2021
**Ragwort**	-	-	−0.93	−0.05
**Other Herbs**	−0.81	−0.85	0.54	−0.48

**Table 6:** Number of flowers of ragwort, other herbs, and grasses counted in botanical plots in both years. n.s. = not significant.

		Number of Flowers/Inflorescences			
Pen (no.)	Plots (*n*)	Interval	Grasses	Ragwort	Other Herbs
1	6	1 May–14 June 2020	1145	0	826
2	6	14 June–24 July 2020	1428	43	1548
3	6	24 July–4 September 2020	792	6	962
4	6	4 September–20 October 2020	0	3	104
**Total**			3365	52	3440
6	6	30 April–14 June 2021	820	0	1249
7	6	14 June–26 July 2021	795	51	1459
8	6	26 July–6 September 2021	14	17	587
9	6	6 September–18 October 2021	3	41	190
**Total**			1632	109	3485
***p***			<0.01	<0.01	n.s.

**Table 7:** Mean coverage (%) of the top 10 grasses and herbs according to the Londo method in 2020 and 2021.

**Top 10 Grasses 2020**			**Top 10 Grasses 2021**		
	**Species**	**Mean Cover (%)**		**Species**	**Mean Cover (%)**
1.	*Agrostis stolonifera*	38.33	1.	*Agrostis capillaris*	20.67
2.	*Agrostis capillaris*	32.44	2.	*Holcus lanatus*	15.67
3.	*Cynosurus cristatus*	17.24	3.	*Lolium perenne*	14.32
4.	*Poa spec.*	14.40	4.	*Bromus hordeaceus*	4.44
5.	*Holcus lanatus*	14.08	5.	*Festuca rubra*	4.15
6.	*Anthoxanthum odoratum*	12.75	6.	*Anthoxanthum odoratum*	4.00
7.	*Alopecurus pratensis*	12.67	7.	*Dactylis glomerata*	3.83
8.	*Lolium perenne*	11.11	8.	*Poa spec.*	3.58
9.	*Carex hirta*	8.46	9.	*Alopecurus pratensis*	2.63
10.	*Bromus hordeaceus*	8.17	10.	*Cynosurus cristatus*	1.32
**Top 10 Herbs 2020**			**Top 10 Herbs 2021**		
	**Species**	**Mean Cover (%)**		**Species**	**Mean Cover (%)**
1.	*Senecio jacobaea*	21.33	1.	*Cirsium arvense*	14.55
2.	*Trifolium repens*	13.24	2.	*Senecio jacobaea*	13.60
3.	*Plantago lanceolta*	13.08	3.	*Potentilla anserina*	7.00
4.	*Ranunculus acris*	11.72	4.	*Trifolium repens*	6.39
5.	*Ranuculus repens*	10.74	5.	*Plantago lanceolata*	6.30
6.	*Cerastium holosteoides*	7.79	6.	*Achillea millefolium*	5.50
7.	*Cirsium arvense*	7.63	7.	*Ranunculus repens*	4.33
8.	*Lotus corniculatus*	7.00	8.	*Hypochaeris radicata*	4.13
9.	*Veronica serpyllifolia*	5.41	9.	*Veronica chamaedrys*	3.15
10.	*Rumex × pratensis*	5.38	10.	*Cerastium holosteoides*	2.31

## Data Availability

Data are contained within the article.
